# Electroencephalography Based Analysis of Working Memory Load and Affective Valence in an N-back Task with Emotional Stimuli

**DOI:** 10.3389/fnhum.2017.00616

**Published:** 2017-12-19

**Authors:** Sebastian Grissmann, Josef Faller, Christian Scharinger, Martin Spüler, Peter Gerjets

**Affiliations:** ^1^LEAD Graduate School, University of Tübingen, Tübingen, Germany; ^2^Laboratory for Intelligent Imaging and Neural Computing, Columbia University, New York, NY, United States; ^3^Leibniz-Institut für Wissensmedien, Multimodal Interaction Lab, Tübingen, Germany; ^4^Wilhelm-Schickard-Institute for Computer Science, University of Tübingen, Tübingen, Germany

**Keywords:** electroencephalography (EEG), working memory, affective valence, emoback, IAPS

## Abstract

Most brain-based measures of the electroencephalogram (EEG) are used in highly controlled lab environments and only focus on narrow mental states (e.g., working memory load). However, we assume that outside the lab complex multidimensional mental states are evoked. This could potentially create interference between EEG signatures used for identification of specific mental states. In this study, we aimed to investigate more realistic conditions and therefore induced a combination of working memory load and affective valence to reveal potential interferences in EEG measures. To induce changes in working memory load and affective valence, we used a paradigm which combines an N-back task (for working memory load manipulation) with a standard method to induce affect (affective pictures taken from the International Affective Picture System (IAPS) database). Subjective ratings showed that the experimental task was successful in inducing working memory load as well as affective valence. Additionally, performance measures were analyzed and it was found that behavioral performance decreased with increasing workload as well as negative valence, showing that affective valence can have an effect on cognitive processing. These findings are supported by changes in frontal theta and parietal alpha power, parameters used for measuring of working memory load in the EEG. However, these EEG measures are influenced by the negative valence condition as well and thereby show that detection of working memory load is sensitive to affective contexts. Unexpectedly, we did not find any effects for EEG measures typically used for affective valence detection (Frontal Alpha Asymmetry (FAA)). Therefore we assume that the FAA measure might not be usable if cognitive workload is induced simultaneously. We conclude that future studies should account for potential context-specifity of EEG measures.

## Introduction

### Investigating Complex User States with the Electroencephalogram

In recent years, there has been increased interest to use the electroencephalogram (EEG) in the context of human-machine interaction (Frey et al., 2013). However, most studies using the EEG to measure mental states focus on very specific states like working memory (Klimesch, 1999) or affective valence (Ahern and Schwartz, 1985), which are investigated in well controlled lab environments. Therefore, the indicators used in such studies might not provide robust measurements outside the lab, since real world environments tend to evoke much more complex and multidimensional mental states that involve different cognitive, emotional and motivational components (Gerjets et al., 2014). Furthermore, many measures used to infer mental states from the ongoing EEG are known to have many-to-many relations, meaning that several physiological variables are associated with multiple psychological elements (Fairclough, 2009). Hence, it is necessary to systematically investigate the relationship between different mental states and the different EEG measures that are widely used in neuroscientific studies, to investigate if such measures can be used outside the lab. In this article we systematically investigate the relation between two types of mental states that are widely used in the context of human-machine interaction, namely working memory load (e.g., Spüler et al., 2016) and affective valence (e.g., De Smedt and Menschaert, 2012), to study the interaction of the brain responses typically associated to these mental states.

### Working Memory Load

There are many different ways to induce mental states characterized by high levels of working memory load (Wilhelm et al., 2013). One way to induce working memory load that has been widely used in the context of cognitive neuroscience is the N-back task (Kirchner, 1958; Scharinger et al., 2015). The N-back task is a continuous performance task where subjects are presented with a series of stimuli and have to indicate whether the current stimulus is identical to the stimulus presented N-steps before, or nor. Hence, the load factor “N” allows to adjust the difficulty of the task, thereby manipulating working memory load.

Working memory load is commonly defined as the interplay of controlled attentional processes and short term memory structures that handle different representational codes via various temporal storage components (Baddeley, 2003, 2012). The central attentional control system is assumed to be mainly located in frontal regions of the brain like the dorsolateral prefrontal cortex, while content in short term memory is thought to be maintained via parietal brain areas like the intraparietal sulcus (Klingberg, 2009; Scharinger et al., 2015). Accordingly, increases in mental workload usually result in increased frontal theta activity as well as decreased parietal alpha activity (Gevins et al., 1995; Klimesch, 1999; Smith and Gevins, 2005). However, previous research has shown that other mental states can also have an effect on measures used for workload detection. Roy et al. (2013), for example, induced fatigue and found that with increasing time on task the discriminability of working memory load was decreased.

### Affective Valence

There are multiple ways to induce affective experiences in lab settings (Gerrards-Hesse et al., 1994). One effective way is the use of affective picture stimuli. The most prominent picture database is the International Affective Picture System (IAPS; Lang et al., 1997) which comprises a large set of standardized and emotionally evocative color photographs. All stimuli of the database have been rated along the dimensions of valence and arousal as described in the two-dimensional circumplex model of emotion (Russell and Pratt, 1980).

The valence dimension reflects the pleasantness of a situation and ranges from sadness to happiness. The arousal dimension reflects the responsiveness of the organism and ranges from sleep to frenzied excitement. Several studies have used the EEG to study affective states in the past Olofsson et al. (2008) and Kim et al. (2013). While there are many different approaches to infer affective valence in the EEG, some even using right hemispheric activity in the beta (Rowland et al., 1985) or gamma band (Müller et al., 1999), the two most widely used EEG measures to infer affective states are the late positive potential (LPP) and the so-called Frontal Alpha Asymmetry (FAA). The LPP is an EEG feature in the time domain and represents a positive deflection in the ERP-curve, reflecting the activity related to the arousal dimension (Schupp et al., 2000). The FAA represents the individual hemispheric contributions which is related to the affective valence dimension (Ahern and Schwartz, 1985; Tomarken et al., 1990; Huang et al., 2012). Increased right frontal activity is an indicator of a mental state characterized by negative affective valence. Usually, this measure is used during resting (with closed eyes) or passive viewing conditions (Davidson, 1992). Interestingly, previous work has shown that working memory load can result in lateralized activity as well. For instance, a study by Baldwin and Penaranda (2012) used several tasks to induce mental workload and found more left hemispheric activity during increased cognitive load. This might result in potential interferences between affective valence and working memory load in the FAA measure. Analyzing this type of potential interference between working memory load and affective valence is the main goal of this article.

### Investigating Interactions between Working Memory Load and Affective Valence: The Emoback Task

Previous research on the use of neural signatures of mental states has largely ignored the problem of potential interference between working memory load and affective experiences. Only one recent study addressed the effect of an affective experience on the automatic identification of working memory load. In this study, Mühl et al. (2014) used an N-back task to manipulate working memory load while social stress was induced with a stress-induction protocol based on the Trier Social Stress Task (Kirschbaum et al., 1993). The authors attempted to detect mental workload during stress using features from the frequency domain as well as the time domain. They concluded that it is possible to transfer methods across affective contexts, but only with diminished performance. However, these results are limited to one specific affective context (social stress). Furthermore, the authors were focusing on the identification of working memory load alone. The current article wants to extend these findings using a more general affective response and also account for the potential interference of cognitive and affective components.

In order to collect suitable data for this objective, we used a combination of an N-back task with a standard affect induction, called the emoback task. Interactions between cognitive and affective processes in the EEG have been previously investigated with the affective flanker task (Alguacil et al., 2013). However, the affective flanker task can be seen as a simple stimulus-response task that only requires perceptual inhibition and therefore does not represent a genuine working memory task. The emoback task does involve memory components and, to our knowledge, has only been used twice in combination with the EEG. First, a study by MacNamara et al. (2011) used the emoback task with affective pictures as distractors and found that the LPP was modulated not only by affective responses towards emotional pictures, but also by working memory load. Second, a study by Kopf et al. (2013) used a N-back task with emotional words and recorded data from EEG and fNIRS. They found more errors during the negative condition, especially for high task difficulty. An ERP-analysis also revealed that the LPP is influenced by the difficulty in the working memory task and that this influence is further modulated by affective valence. However, both these studies used the LPP, as feature commonly associated with the arousal dimension. We want to investigate affective responses related to the valence dimension, which allows to make more general discriminations of affective states into positive and negative states. Moreover, the study by MacNamara et al. (2011) only used affective stimuli as distractors, while we want to use a paradigm that inherently activates cognitive as well as affective processes. Finally, while the study by Kopf et al. (2013) used emotional words to induce affective reactions, we assume that affective pictures can elicit stronger affective reactions. We therefore decided to use an emoback task with affective pictures from the IAPS database to investigate potential interferences between working memory load and affective valence using frontal theta activity, parietal alpha activity as well as the FAA.

In previous analyses of the same dataset (Grissmann et al., in press), we found that classification of working memory load under affective valence can result in classification accuracies above 70%, which can be further improved via data integration over time. However, we also found that positive as well as negative valenced affective contexts led to decreased classification accuracies, when compared to a neutral affective context. Additionally, classifiers failed to generalize across affective contexts, which highlighted the need to better understand the interactions between working memory load and affective valence in such a context.

### Research Questions and Hypotheses

We investigated the influence of working memory load and affective valence on subjective measures, behavioral measures as well as the corresponding EEG measures. Furthermore, we investigated potential interferences between cognitive processes and affective processes as reflected in EEG measures used to infer working memory load and EEG measures used to infer affective valence.

More specifically, we investigated potential effects of load levels in the emoback task on subjective ratings, accuracies and reaction times, which might also be reflected in the corresponding EEG measures.

Additionally, we investigated potential effects of the affective valence inductions in the emoback task on subjective measures, behavioral measures as well as EEG measures.

Beyond the main effects of working memory load and affective valence on behavioral measures and EEG measures, we also analyzed whether EEG measures used for mental workload detection are sensitive to different affective contexts and whether EEG measures used to infer affective valence are sensitive to working memory load.

## Materials and Methods

### Sample

For this study, we collected data from 27 female subjects. Female subjects were used in this study because they tend to show stronger reactions toward affective stimuli (Lang et al., 1993) and also exhibit more stable responses (Ahern and Schwartz, 1985). Three subject were removed due to the low quality of the signals. All of the participants were university students, aged above 18 years (mean: 23.0 years; range: 19–32 years), right handed and had no blood phobia to avoid extreme responses toward the experimental stimuli. All subjects provided written informed consent and were paid 20 € for participation in the experiment. The study was approved by the ethic committee of the Knowledge Media Research Center Tuebingen.

### Recording of Physiological Data

Sixty channels of EEG were recorded using an ActiCHamp amplifier and active Ag/Cl-electrodes (Brainproducts GmbH, Gilching, Germany). Electrodes were placed according to the extended 10-20 system. The electrooculogram (EOG) was recorded with four EEG electrodes located at the left and right canthi as well as above (channel Fp1) and below the left eye. All channels were referenced to channel FCz. Impedances were kept below 10 kΩ. The data were sampled at 1 kHz. This electrode layout was chosen to allow for potential source localization approaches. For later processing the data was downsampled to 250 Hz. During the recordings subjects were instructed to sit in a relaxed posture to avoid artifact contamination of the data.

### Preprocessing

To automatically reject time windows that are contaminated by artifacts, a time window was removed if channel power at more than six channels exceeded five times the standard deviation. After computing independent components using the CUDAICA implementation (Raimondo et al., 2012) of the Infomax independent component analysis (Bell and Sejnowski, 1995), eye movement artifacts were removed (reduced) using the ADJUST approach (Mognon et al., 2011) which is implemented as plug-in in the EEGLAB toolbox (Delorme et al., 2011). Finally the EEG signal was bandpass filtered between 1 Hz and 45 Hz using two-way least-squares finite impulse response filtering.

### Stimuli

For the positive, neutral and negative valence conditions we selected 96 stimuli each. The stimuli were taken from the IAPS database (Lang et al., 1997) and selected based on the valence and arousal ratings provided with the database. Valence and arousal ratings are usually confounded, meaning that stimuli with strong (positive or negative) valence ratings usually also have high arousal ratings. To improve discriminability between affective conditions we made sure that stimuli for the positive condition had the highest valence ratings while stimuli for the negative conditions had the lowest valence ratings. Furthermore, stimuli for the neutral condition were selected based on the lowest arousal ratings. All selected stimuli had a quadratic shape and were centrally presented on a standard 23 inch display. To avoid unnecessary eye-movements the size of the stimuli was scaled to fill 60% of the height of the display.

### Study Design and Block Structure

**Figure 1:**
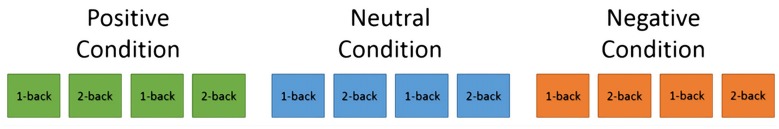
Illustration of EMOBACK study design: the three affective valence conditions were presented grouped and permutated across the whole sample. Each affective condition consisted of four workload blocks, resulting in 12 blocks for each subject. Workload blocks were presented in an alternating fashion and balanced across the whole sample.

The affective valence conditions were presented in groups of four trial blocks with identical affective valence. The affective valence conditions being either positive, neutral or negative. Load levels of the emoback task were alternated block wise. The load factor of the emoback task had two levels, one-back and two-back. We avoided the use of a zero-back condition, because this would require to repeatedly use the same affective target stimuli. This repetition of the same stimuli might have resulted in affective habituation with regard to the target stimuli, thereby diminishing the affect induction (Leventhal et al., 2007). We also avoided a 3-back condition because some studies showed that this load level can lead to task disengagement (Ayaz et al., 2007). Target response hand as well as starting load level of the emoback task were balanced across the subject sample. See Figure 1 for an illustration of the study design.

**Figure 2:**
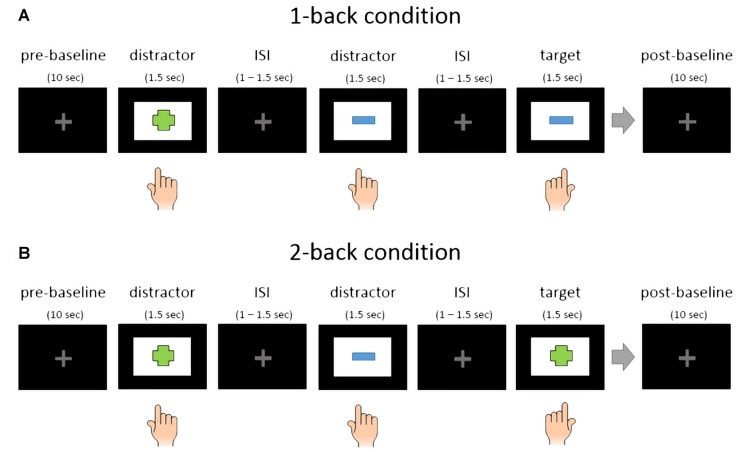
Schematic representation of EMOBACK block structure. **(A)** 1-back condition. **(B)** 2-back condition. Hand symbols indicate key press. Left hand press was used for target stimuli and right hand press for distractors in this example. The simple shapes in this illustration only serve as an example.

All participants performed 12 blocks in total. Each block started with a 10 s baseline where subjects were instructed to relax and visually fixate a centrally presented light gray fixation cross. After the baseline the first trial started with a stimulus presentation. There were 72 trials in one block, consisting of 24 target stimuli and 48 distractors. The 24 target stimuli were randomly selected for each subject from 96 unique stimuli (four blocks with 24 target stimuli per affective condition). Targets and distractors were sometimes interleaved in the 2-back condition, meaning that two target stimuli could appear right after each other. Here is an example: Distractor (e.g., table), distractor (e.g., chair), target (table) and target (chair). After the last trial, another baseline phase, also with a duration of 10 s, was recorded. Between blocks there were short brakes between 1 min and 3 min. See Figure 2 for an illustration of the block structure.

### Subjective Measures

After each run, subjects were asked to rate their subjective experience of the last run. Subjective experience of working memory load was measured using one (modified) item taken from the NASA task load index (Hart and Staveland, 1988). The item asked participants how cognitively demanding they experienced the last experimental run. The rating scale ranged from 0 (absolutely no mental demand) to 100 (highest possible mental demand).

Emotional experiences are commonly judged with the help of rating scales. The most widely used is a visual analog scale called the Self-Assessment Manikin (SAM; Bradley and Lang, 1994). It enables fast and reliable judgments about the current emotional state.

### Trial Structure

Each trial started with a stimulus presentation phase of 1500 ms. The duration of the stimulus presentation was selected based on tests using pilot subjects and should ensure that enough trials can be recorded to get reliable estimates for the EEG measures used and the desired affective responses are evoked. During the stimulus presentation phase the subjects had to indicate if the current stimuli was a target (i.e., identical to the stimulus one step before in the 1-back condition and two steps before in the 2-back condition) or if the current stimulus was a distractor. Left and right control keys of a standard keyboard were used as inputs. The subjects were instructed to react as quickly as possible to ensure an effect in performance measures. Between stimuli there were interstimulus intervals (ISI) of 1500 ms duration including one to 500 ms of jitter at the end of the ISI to avoid periodic responses in the EEG data. During the ISI the same gray crosshair as in the baseline conditions was presented on the screen.

From psychological perspective, the N-back task may conceptually be divided into two phases. The first phase starts with the stimulus presentation and ends after the stimulus response. In this phase we assume that subjects had to perform a simple matching task by comparing the current stimulus with the stimulus stored in short term memory. In the second phase, ranging from the stimulus response to the next stimulus onset we assume that multiple executive functions are required for a correct response. Inhibition, shifting and updating represent the core executive functions as identified by Miyake et al. (2000). Inhibition is seen as the ability to suppress automatic responses that might arise during task processing. Shifting is referring to the concept of cognitive flexibility, making us capable to switch between different tasks. Updating refers to the continuous monitoring and changing of content in working memory. Content in short term memory needs to be updated via inhibition of the last stimuli in the stimuli list. Furthermore, the participants need to switch from a simple matching task to a working memory task and back. We therefore assumed that the second phase was more relevant for the measurement of working memory load. Concerning the affective reaction we first assumed that it would be stronger right after stimulus onset, since the stimulus was fresh. However, pilot measurements revealed that subjects tend to focus first on the response toward the stimuli and only then direct their attention toward the affective content of the presented stimuli. We therefore decided to focus our analyses on the second phase. Additionally, we wanted to avoid contamination of the data due to muscular artifacts which originate from the keyboard input.

### Analysis

The window of analysis was 1400 ms wide. It started 1100 ms after stimulus onset to exclude post-motor responses in the EEG and ended 2500 ms after stimulus onset. The time window also included 1000 ms of the ISI.

All spectra for the EEG analysis were computed using the Welch method (Welch, 1967) implemented in the EEGLAB toolbox (Delorme and Makeig, 2004). Theta bands were computed between 4 Hz and 7 Hz and alpha bands were computed between 8 Hz and 12 Hz. Power of the frontal (AFz, Fz) and parietal (CPz, Pz, POz) electrodes was averaged. Power for FAA computation was averaged across channels AF3, F3 and FC1 for the left hemisphere and across channels AF4, F4 and FC2 for the right hemisphere. We followed the approach from Allen et al. (2004) and computed FAA as difference score (see Equation 1).

Equation 1: Frontal Alpha Asymmetry Index (Allen et al., 2004).

FAA=right alpha power−left alpha power

To evaluate the influence of affective valence and working memory load on the EEG, we performed repeated measures analysis of variances (ANOVAs) with two factors. The first factor was affective valence with three levels (positive, neutral and negative). The second factor was working memory load with two levels (1-back and 2-back). All analyses were conducted at the group level.

## Results

### Subjective Measures—Working Memory Load

**Figure 3:**
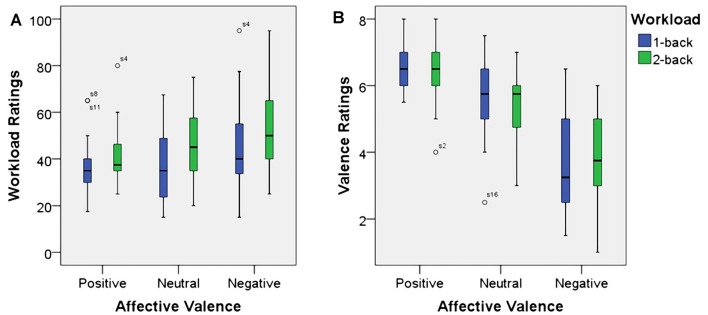
Box plots showing median values for workload ratings **(A)** and affective valence ratings **(B)**. Blue boxes show working memory load ratings **(A)** and affective valence ratings **(B)** for the 1-back conditions. Green boxes show working memory load ratings **(A)** and affective valence ratings **(B)** for the 2-back conditions. Median values are indicated by black horizontal lines within the boxes. Top and bottom borders of the boxes represent the middle 50% of the data. Whiskers represent the smallest and largest values not classified as outliers (between 1.5 and 3 times the height of the boxes) or extreme values (more than three times the height of the boxes). Circles indicate outliers.

As expected, using workload ratings as dependent variable, we found a significant main effect for working memory load, *F*_(1,23)_ = 22.7, *p* < 0.001, *η*^2^ = 0.50. Higher working memory load resulted in the increased subjective experience of working memory load. Additionally, there was a significant main effect for affective valence, *F*_(2,46)_ = 11.6, *p* < 0.001, *η*^2^ = 0.34. A *Post hoc* test using the Šidák correction revealed that the negative valence condition resulted in an increase of subjective working memory load when compared to the positive valence condition (*p* < 0.001) as well as the neutral valence condition (*p* < 0.02). There was no significant interaction between affective valence and working memory load, *F*_(2,46)_ = 0.8, n.s. Box plots showing the different conditions can be seen in Figure 3A.

### Subjective Measures—Affective Valence

Since, this manuscript was focusing on the effect of the experimental manipulation on the EEG measure used to infer affective valence, we do not report results from the arousal dimension here. As anticipated, using the affective valence ratings as dependent variable, there was a significant main effect for affective valence, *F*_(1.4,33)_ = 55.7, *p* < 0.001, *η*^2^ = 0.71. Decreased affective valence due to the emotion induction resulted in decreased subjective valence ratings. The neutral condition resulted in more positive valenced ratings than the negative condition (*p* > 0.001) and more negative valenced ratings than the positive condition (*p* > 0.01). However, there was neither a significant main effect for working memory load, *F*_(1,23)_ = 0.1, n.s., nor was there a significant interaction between affective valence and working memory load, *F*_(2,46)_ = 1.0, n.s. Median values for all conditions are shown via box plots in Figure 3B.

### Behavioral Performance Measures—Accuracies

**Figure 4:**
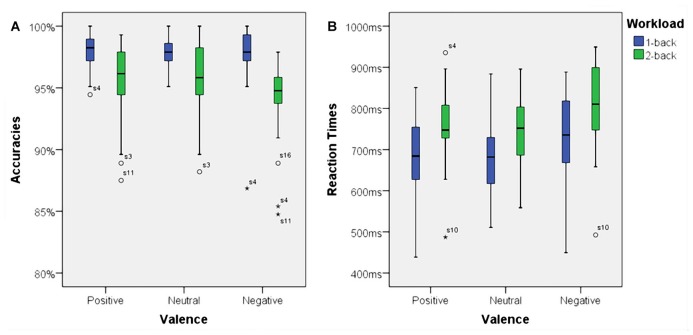
Box plots showing median values for accuracies and reaction times. Blue boxes show accuracies **(A)** and reaction times **(B)** for the 1-back conditions. Green boxes show accuracies **(A)** and reaction times **(B)** for the 2-back conditions. Median values are indicated by black horizontal lines within the boxes. Top and bottom borders of the boxes represent the middle 50% of the data. Whiskers represent the smallest and largest values not classified as outliers (between 1.5 and 3 times the height of the boxes) or extreme values (more than three times the height of the boxes). Circles indicate outliers and stars show extreme values.

Using accuracies as dependent variable we found a significant main effect for working memory load, *F*_(1,23)_ = 32.70, *p* < 0.001, *η*^2^ = 0.59. Higher working memory load resulted in decreased accuracies. Additionally, there was a significant main effect for affective valence, *F*_(2,46)_ = 3.73, *p* < 0.035, *η*^2^ = 0.14. A *Post hoc* test using the Šidák correction revealed that the negative condition resulted in a decreased accuracy when compared to the positive condition (*p* < 0.03), but not when compared to the neutral condition (n.s.). Interestingly, there was a significant interaction between affective valence and working memory load, *F*_(2,46)_ = 3.68, *p* < 0.035, *η*^2^ = 0.14. The negative condition had a negative impact on accuracy, but only under high working memory load. Box plots showing the different conditions can be seen in Figure 4A.

### Behavioral Performance Measures—Reaction Times

There was a significant main effect for working memory load, *F*_(1,23)_ = 60.53, *p* < 0.001, *η*^2^ = 0.73. Higher load resulted in longer reaction times. Moreover, there was a significant main effect for affective valence, *F*_(2,46)_ = 10.94, *p* < 0.001, *η*^2^ = 0.32. The negative condition resulted in longer reaction times than the positive condition (*p* < 0.01) as well as the neutral (*p* < 0.005) condition. However, there was no significant interaction between affective valence and working memory load, *F*_(2,46)_ = 1.02, n.s. Median values for all conditions are shown via box plots in Figure 4B.

### EEG Working Memory Load Measures

#### Frontal Theta

**Figure 5:**
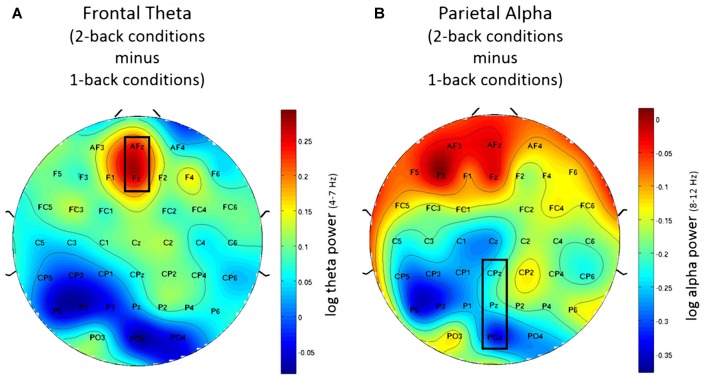
Topographic plots showing difference in electroencephalography (EEG) workload measures between 2-back and 1-back conditions. **(A)** Frontal theta for the post-motor time window showing increased frontal theta activity for the 2-back conditions. **(B)** Parietal alpha for the Post motor time window showing decreased in parietal alpha power for the 2-back conditions. Nose is at the top. Values are averaged across all three affective conditions. Electrodes used for analysis are marked with black rectangles.

**Figure 6:**
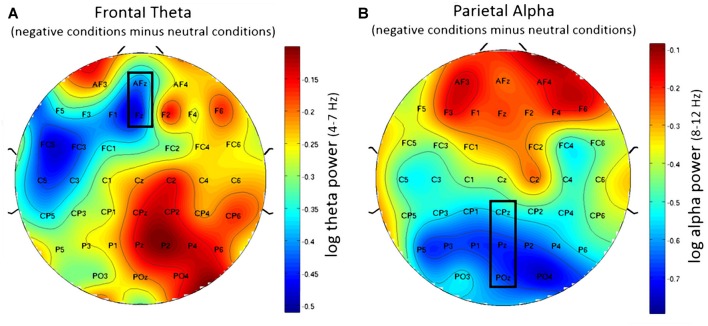
Topographic plots showing difference in EEG workload measures between negative and neutral conditions. **(A)** Frontal theta showing decreased frontal theta activity for the negative conditions when compared to the neutral conditions. **(B)** Parietal alpha showing widespread decrease in parietal alpha power for the negative conditions when compared to the neutral conditions. Nose is at the top. Values are averaged across both N-back levels. Electrodes used for analysis are marked with black rectangles.

**Figure 7:**
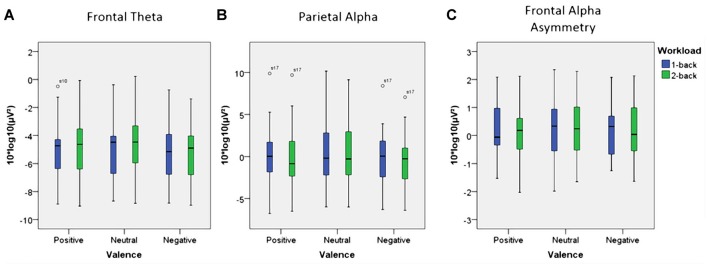
Box plots showing median values for frontal theta activity, parietal alpha activity and Frontal Alpha Asymmetry (FAA). Blue boxes show frontal theta activity **(A)**, parietal alpha activity **(B)** and FAA **(C)** for the 1-back conditions. Green boxes show frontal theta activity **(A)**, parietal alpha activity **(B)** and FAA **(C)** for the 2-back conditions. Median values are indicated by black horizontal lines within the boxes. Top and bottom borders of the boxes represent the middle 50% of the data. Whiskers represent the smallest and largest values not classified as outliers (between 1.5 and 3 times the height of the boxes) or extreme values (more than three times the height of the boxes). Circles indicate outliers. Please note the strong variability in the data due to large inter-individual differences.

Using frontal theta activity as dependent variable there was a significant main effect for working memory load, *F*_(1,23)_ = 9.00, *p* < 0.01, *η*^2^ = 0.28. Frontal theta power was higher in the 2-back conditions. Figure 5A illustrates this effect. Interestingly, there was a significant main effect for affective valence, *F*_(2,46)_ = 8.28, *p* < 0.001, *η*^2^ = 0.27. A *Post hoc* test using the Šidák correction revealed that frontal theta power was lower in the negative condition, when compared to the neutral condition (*p* < 0.005) as well as the positive condition (*p* < 0.01). Figure 6A shows a topographic plot displaying this frontal theta effect. However, there was no significant interaction between affective valence and working memory load, *F*_(2,46)_ = 0.87, n.s. Median values for all conditions can be seen in Figure 7A.

#### Parietal Alpha

Using parietal alpha activity as dependent variable we found a significant main effect for working memory load, *F*_(1,23)_ = 4.22, *p* = 0.05, *η*^2^ = 0.16. Higher working memory load resulted in decreased parietal alpha power. This effect can be seen in Figure 5B. Interestingly, there was also a significant main effect for the factor affective valence in the post-motor window, *F*_(2,46)_ = 5.27, *p* < 0.01, *η*^2^ = 0.19. The negative condition exhibited lower parietal alpha power than the neutral condition (*p* < 0.03), but not lower than the positive condition (n.s.). Figure 6B shows a topographic plot displaying this parietal alpha effect. There was no significant interaction between affective valence and working memory load, *F*_(2,46)_ = 1.52, n.s. Figure 7B shows box plots for all conditions.

### EEG Affective Valence Measures—Frontal Alpha Asymmetry

Unexpectedly, there were no significant main effects with regard to FAA. Neither for affective valence, *F*_(2,46)_ = 1.87, n.s. nor for working memory load, *F*_(1,23)_ = 0.96, n.s. Finally, there was also no significant interaction between affective valence and working memory, *F*_(2,46)_ = 0.08, n.s. Median values for all conditions are summarized in Figure 7C.

## Discussion

Using the emoback paradigm, we found that increased working memory load had a negative impact on task performance, as reflected in decreased accuracies and increased reaction times. These effects were also reflected in corresponding EEG measures. Increased working memory load was accompanied by increases in frontal theta activity as well as decreases in parietal alpha activity.

We found that negative affective valence had a negative impact on accuracies as well as reaction times. Interestingly, measures used for working memory load estimate also appeared sensitive to changes in affective valence. In contrast to that, FAA, a measure typically used to infer affective valence, did not show any effects in our paradigm, neither with regard to affective valence, nor with regard to increases in working memory load. In the following sections we will discuss these results in detail.

### Subjective Measures

As expected, we found that increased working memory load, due to an increase in the load factor of the emoback task, resulted in higher subjective working memory load ratings. This finding is reassuring, especially since some subjects reported difficulties judging the working memory load with regard to the difficulty of the emoback task. Some subjects even reported that they experienced the 1-back conditions as more demanding, due to their monotonous nature.

As hypothesized, we also found a significant effect of affective valence on ratings from the valence dimension of the SAM. Notably, the successful induction of positive affective valence shows that the emoback paradigm worked in the intended way. The induction of positive emotions can be very difficult to achieve in laboratory environments, since positive emotions usually arise in a specific context which is difficult to represent in a single picture (Kim and Hamann, 2007).

### Behavioral Performance Measures

Our analyses revealed that increased working memory load induced via the emoback task did result in decreased performance. The 2-back conditions had reduced accuracy as well as increased reaction times. We also found that inducing negative affective valence had detrimental effects on accuracies as well as reaction times. Similar results have been found in a study by Passarotti et al. (2011). The authors used face stimuli in an affective N-back task and found slower reaction times for angry faces. These negative effects of affective stimuli on cognitive processing might be explained within the theory of hot and cold cognition, concepts related to executive function (Zelazo and Müller, 2002). Hot cognition refers to a process where a persons’ thinking is influenced by their affective state (Brand, 1986), while cold cognition is more based on rational thinking and critical analysis (Roiser and Sahakian, 2013). Hot cognition seems to be able to overpower cold cognition in certain situations and has been shown to impair decision making (Huijbregts et al., 2008). Processing of negative stimuli can divert cognitive resources from the primary task, and thereby lead to decreased performance. A study by Levens and Gotlib (2010) found that strongly valenced stimuli tend to stay longer active in working memory, which might interfere with different core executive functions necessary during the emoback task (Miyake et al., 2000). In the emoback task, subjects constantly needed to update content in working memory by replacing (inhibition) previous stimuli. Additionally, the subjects seemed to need to shift between these updating tasks and a rather simple identity-matching task. Negative stimuli might catch the attention, slowing down reorganization of stimuli in working memory and thereby impairing task performance.

Interestingly, we also found an interaction for accuracy between working memory load and affective valence. Negative affective valence resulted in decreased accuracies, but only during increased working memory load. These results are in line with findings from the study by Kopf et al. (2013) who found more errors for the difficult task during the negative condition using an affective word N-back task. These findings seem to indicate that both, cognitive and affective processing, compete for limited cognitive resources and this dual strain on mental resources leads to decreased performance. Similar results have also been found in the study by MacNamara et al. (2011). The authors used emotional pictures as distractors in an emoback task and found that the emotional content of negative valenced stimuli increased the negative impact of working memory load on performance. These findings might be explained using the capacity model from Ellis and Ashbrook (1989). Their model assumes that there is a limited pool of attentional resources that can be used to complete a certain task. Affective states can influence the allocation of available attentional resources toward the task, thereby potentially impairing task performance. However, based on performance measures alone, we can only vaguely assume how negative valence impaired task performance.

### EEG Working Memory Load Measures

#### Frontal Theta Activity

Increased working memory load was reflected in EEG measures as well. Frontal theta activity was increased during high working memory load. Interestingly, the negative affective valence condition exhibited decreased frontal theta after the motor response. If negative affective valence would impair performance via the production of additional working memory load, one would expect to find increased frontal theta activity during negative affective valence. However, the decreased frontal theta activity under negative affective valence indicates that the detrimental effect on performance likely has different reasons than the performance decrease under high working memory load. Accordingly, we assume that the negative affective valenced stimuli interfered with task processing through a reduction of activity in the frontal attentional control network.

#### Parietal Alpha Activity

Our analyses revealed that parietal alpha activity was decreased under high working memory load (2-back) condition. We also found that the parietal alpha activity was reduced for the negative condition. An fMRI study by Rämä et al. (2001) used different affective voices and found similar results. The authors discovered that the parietal cortex is bilaterally involved in active maintenance of emotional content. Affective stimuli are more salient and usually carry more relevant information (Carretié, 2014). We therefore assume that negative valenced stimuli result in stronger processing in the storage areas of the parietal cortex, which seems to be reflected through increased parietal activity (i.e., reduced alpha power).

### EEG Affective Valence Measures—Frontal Alpha Asymmetry

Unexpectedly, we did not find any effect using the FAA measure. We used the FAA measure during the induction of working memory load, while most other studies used brain laterality responses during rest or passive viewing (Ahern and Schwartz, 1985; Tomarken et al., 1992; Lin et al., 2009; Huang et al., 2012; Ramirez and Vamvakousis, 2012). Use of the FAA in combination with other tasks is further complicated because the FAA is known to be also influenced by other factors like seating position and working memory load (Briesemeister et al., 2013). A study by Baldwin and Penaranda (2012) found that increased task difficulty resulted in increased left frontal activity. It appears that the negative condition increased task difficulty, since it did impair task performance. Negative affective valence in passive conditions usually results in increased right frontal activity. It is therefore conceivable that both, affective and workload related, processes acted on the same FAA measure, but in opposing directions. This could mean that both effects canceled each other out and thereby masked any potential effects.

### Limitations and Outlook

We did not find any effects using the FAA measure. We assume that was due to the motor response required in our paradigm. It would be interesting to test this assumption by developing a study design that contrasts an active condition with a passive viewing condition. However, it could also be possible, that the paradigm did not induce sufficient stress to induce changes in the FAA measure. A study by Goodman et al. (2013) used a working memory task and concurrently induced stress. The authors found that changes in the FAA measure (as indicated by left frontal activity) can be used to infer emotional processing, but only when the emotional induction reaches a certain intensity. Future studies could try to use other ways to induce emotional reactions along the valence dimension, like short video clips.

Additionally, our intent to induce the strongest possible valence effect had the drawback that the affective conditions also differed concerning the arousal dimension. This meant that the negative conditions were also experienced as more arousing. Since even this valence induction did not produce a valence effect that could be measured with the FAA, we recommend that future studies should control for arousal as well as other dimensions like affective dominance.

Furthermore, we based our choice of the window used for analysis on our understanding of the existent literature. However our assumptions need further support and therefore future studies should investigate the different sub-processes that contribute to the n-back task and their temporal evolution in greater detail.

While we decided to focus on the use of a frequency based approach, future approaches could also include different features for the measurement of affective valence and working memory load. One example would be the use of the ERP, since previous studies have already demonstrated that the ERP has some potential in this context (see Olofsson et al., 2008; Brouwer et al., 2012). Another approach could be the use of connectivity measures, which have already been shown to be of use in similar contexts (e.g., Lee and Hsieh, 2014). A study by Martini et al. (2012) has even combined frequency measures, ERP measures and connectivity measures to differentiate neutral from negative pictures.

Future studies should also explore how well these findings generalize to male individuals as well as other stimulus modalities.

Finally, in this study we focused on the interaction between working memory load and affective valence, using established measures that were already used in the context of human-machine interaction. Future studies should further investigate this issue to gain more insight about the processes underlying the results that were extracted from the EEG-recordings. One example for such an integrative approach is the review by Schwabe et al. (2012) that integrates multiple findings into a framework that allows to create hypotheses with regard to specific neural structures. Something which is beyond the methodology used in this study.

## Conclusion

We demonstrated that increased working memory load and negative valenced stimuli can have an effect on performance measures. Additionally, when using established EEG measures we found that increased working memory load can be detected in the EEG, even when affective valence is induced at the same time. However, EEG measures used to infer working memory load were still influenced by negative affective valence. Furthermore, FAA did not prove useful for identification of emotional states when working memory load is induced at the same time. Therefore, future studies should further investigate the context sensitivity and applicability of EEG measures in various contexts to identify the ramifications in which such measures can be used to identify different states and processes.

## Author Contributions

SG is the main author and responsible for all tasks. JF was involved in the planning of the experiment and also provided technical advice. CS and MS helped with general advice. PG is the main coordinator and also involved in all related tasks.

## Conflict of Interest Statement

The authors declare that the research was conducted in the absence of any commercial or financial relationships that could be construed as a potential conflict of interest.
